# Rare Occurrence of Microsatellite Instability in Gastrointestinal Stromal Tumors

**DOI:** 10.3390/medicina57020174

**Published:** 2021-02-18

**Authors:** Joonhong Park, Hae Jung Sul, Jeong Goo Kim

**Affiliations:** 1 Department of Laboratory Medicine, Jeonbuk National University Medical School and Hospital, Jeonju 54907, Korea; miziro@jbnu.ac.kr; 2 Clinical Medicine-Biomedical Research Institute, Jeonbuk National University Hospital, Jeonju 54907, Korea; 3 Department of Pathology, College of Medicine, The Catholic University of Korea, Seoul 06591, Korea; hjsul@cmcdj.or.kr; 4 Department of Surgery, College of Medicine, The Catholic University of Korea, Seoul 06591, Korea

**Keywords:** microsatellite instability, next-generation sequencing, MSICall, somatic mutation profile, gastrointestinal stromal tumor

## Abstract

*Background and Objectives:* This study aimed to objectively determine microsatellite instability (MSI) status using a next-generation sequencing (NGS)-based MSI panel and to resolve the discrepancy regarding whether or not MSI is a rare phenomenon, irrespective of diverse genomic alterations in gastrointestinal stromal tumors (GISTs). *Materials and Methods:* Genomic DNA was subjected to MSI panel sequencing using an Ion AmpliSeq Microsatellite Instability Assay, as well as to cancer gene panel sequencing using an Oncomine Focus DNA Assay. *Results:* All of our GIST patients showed microsatellite-stable (MSS) status, which confirmed that MSI status did not affect the molecular pathogenesis of GIST. The *KIT* gene (79%, 38/48) was the most frequently mutated gene, followed by the *PDGFRA* (8%, 4/48), *PIK3CA* (8%, 4/48), and ERBB2 (4%, 2/48) mutations. *KIT* exon 11 mutant patients were more favorable in responding to imatinib than those with exon 9 mutant or wild-type GISTs, and compared to non-KIT exon 11 mutant GISTs (*p* = 0.041). The NGS-based MSI panel with MSICall confirmed a rare phenomenon of microsatellite instability in GISTs irrespective of diverse genomic alterations. *Conclusion:* Massively parallel sequencing can simultaneously provide the MSI status as well as the somatic mutation profile in a single test. This combined approach may help us to understand the molecular pathogenesis of GIST carcinogenesis and malignant progression.

## 1. Introduction

Microsatellites are repetitive tracts in certain DNA motifs, ranging from one to six or more nucleotide pairs in length [1]. Replication slips are recognized and repaired through the DNA mismatch repair (MMR) system, such as with MSH6, MLH1, MSH2, and PMS2, to conserve cellular homeostasis [2]. Their lengths are altered during DNA replication because microsatellite loci are vulnerable to replication errors [3]. If the MMR system is functional, replication errors are normally fixed and somatic mutations are stacked up. This genomic phenomenon is referred to as microsatellite instability (MSI). In general, the conventional MSI assay—which consists of a polymerase chain reaction (PCR) followed by fragment analysis using capillary electrophoresis and immunohistochemistry (IHC) for MMR proteins—has been widely applied as a gold standard for estimating MSI status. However, these tests require visual interpretation and make it challenging to decide on MSI status with inconclusive data. Discordant results between IHC and the conventional MSI assay have been reported due to various possible situations [3]. Variations in the mutant allele burden with respect to tumor heterogeneity and tumor purity can influence the determination of MSI status [2,4]. Somatic mutations accompanied by microsatellites related to crucial target genes are considered to play a critical role in the evolution of deficient MMR (dMMR) tumors [5]. On the other hand, IHC can be used to identify the expression loss of MMR proteins when deleterious mutations, such as nonsense, frameshift, and splice-site mutations, occur in MMR genes. However, MMR proteins show normal expression, even though pathogenic missense mutations cause functional defects in MMR proteins. In such conditions, IHC shows false negatives for dMMR tumors. Thus, more sensitive and specific diagnostic tests for determining MSI status are required. Now, next-generation sequencing (NGS) analysis allows us to determine MSI status and comprehensive genomic mutation profiles simultaneously. Recently, an NGS-based MSI assay with MSICall has been developed as an alternative assay [6].

MSI is found in up to 20% of sporadic gastric, colorectal, and endometrial cancers, even though it is less frequent in other types of solid tumors [7,8]. The MSI status is a molecular subtype that is especially recognized in gastric cancer [9]. The incidence of gastric cancers with high MSI has been known to be up to 37%, and has been associated with older age, earlier tumor stages, being female, less frequent lymph node metastasis, and intestinal histology [10,11]. Gastric cancers with high MSI are considered as a good prognosis factor, and MSI status may be a useful biomarker for predicting chemotherapy responsiveness for stage II/III gastric cancer [12,13]. However, the occurrence of an MSI phenotype in gastrointestinal stromal tumors (GISTs) has not been thoroughly evaluated, and the clinical implications of MSI status need to be determined.

In this study, we objectively determined MSI status using an NGS-based MSI panel and resolved the discrepancy regarding whether or not MSI is a rare phenomenon, irrespective of diverse genomic alterations in GISTs.

## 2. Materials and Methods

### 2.1. Samples and DNA Extraction

The study protocol was approved by the Institutional Review Board of The Catholic University of Korea and informed consent was obtained from all patients (DC18SESI0113; Date of approval: 13 November 2018). All participants and their parents provided written informed consent for clinical analyses. A total 48 formalin-fixed paraffin-embedded (FFPE) samples of GIST tissues were obtained from Korean patients diagnosed with GISTs at Daejeon St. Mary’s Hospital (Daejeon, Korea). Histological classification from the same tissues was confirmed by a board-certified pathologist. The pathologic diagnosis of GISTs was determined according to a recommendation by the Korean GIST Study Group [14]. In particular, all 48 patients that were positive for c-KIT but negative for S100 and desmin in the IHC analysis were diagnosed with GISTs. FFPE samples that had more than 50% tumor content were analyzed in this study; they were divided into 10 micrometer sections via laser-capture microdissection by a certified pathologist, and were preserved in 1.5 mL Eppendorf tubes. The blade was changed for every tissue block to prevent contamination.

Genomic DNA was isolated from the four or five unstained FFPE sections using the Recover All Total Nucleic Acid Isolation Kit (Thermo Fisher Scientific, Waltham, MA, USA) per the manufacturer’s instructions. Amplifiable genomic DNA was assessed quantitatively using a Qubit dsDNA High-Sensitivity Assay Kit, a Qubit 2.0 fluorometer, and a TaqMan RNase P Detection Reagent Kit (Thermo Fisher Scientific, Waltham, MA, USA); it was considered adequate when the nucleic acid concentration was >10 ng/uL.

### 2.2. Microsatellite Instability Analysis

The genomic DNA was subjected to MSI panel sequencing using an Ion AmpliSeq Microsatellite Instability Assay (ThermoFisher Scientific, Waltham, MA, USA) consisting of a single primer pool, which amplified a total of 76 amplicons to assess the MSI status, as per the manufacturer’s protocol. The libraries for the MSI panel were partially phosphorylated using the FuPa reagent, ligated to different barcode adapters using an Ion Xpress Barcode Adapter 1–48 Kit (ThermoFisher Scientific, Waltham, MA, USA), and purified. The purified libraries were quantified using an Ion Library TaqMan Quantitation Kit (Thermo Fisher Scientific, Waltham, MA, USA). The pooled purified libraries of ten multiplexed tumor DNAs per Ion 318 v2 chip (ThermoFisher Scientific, Waltham, MA, USA) at a concentration of 50 pM were used for chip loading onto an Ion Chef with an Ion PGM Hi-Q Chef Kit (ThermoFisher Scientific, Waltham, MA, USA), and were subsequently sequenced on an Ion Torrent Personal Genome Machine (PGM) using an Ion PGM Hi-Q Sequencing Kit (ThermoFisher Scientific, Waltham, MA, USA). Control DNA of the CEPH was included in the same sequencing run. MSICall, an Ion Torrent Suite software plugin, was used to determine the target sites and to estimate the MSI score of each marker generated from the Ion AmpliSeq Microsatellite Instability Assay. Markers with over 30 mapped and filtered reads were considered to stand for the MSI score. Markers with scores less than 0.5, the marker score threshold, were filtered in to calculate the marker scores and MSI status of each sample, which were interpreted as follows: high MSI, MSI score ≥ 40; microsatellite stable (MSS), <40.

In addition, a conventional MSI assay (Genetree Research, Seoul, Korea) was performed for the same DNA to confirm the MSI status of each GIST using a set of five mononucleotide repeat markers (BAT-25, BAT-26, NR-21, NR-24, and NR-27), as described previously [15]. The PCR products labeled with fluorescent dye were analyzed through capillary electrophoresis using a 3730 XL Genetic Analyzer (Applied Biosystems, Carlsbad, CA, USA), and the MSI status of each GIST was estimated and compared with that of the corresponding normal control (high MSI, ≥2 markers unstable; MSS, no marker unstable) with GeneMapper Software 5 (Applied Biosystems).

### 2.3. Somatic Mutation Profile Analysis

The genomic DNA was subjected to cancer gene panel sequencing using an Oncomine Focus DNA Assay (ThermoFisher Scientific, Waltham, MA, USA) consisting of hotspot mutations of single nucleotide variants (SNVs), as well as insertions and deletions (Indels) of genes that are relevant to the targeted therapy of solid tumors, including *KIT* and *PDGFRA*. Massively parallel sequencing was conducted using the Ion 318 v2 chip and Ion PGM Hi-Q Sequencing Kit on the Ion Torrent PGM (ThermoFisher Scientific, Waltham, MA, USA), and the “Oncomine™ Focus-520-w2.4-DNA-Single Sample” automatic workflow in Ion Reporter was applied to detect and annotate the somatic mutations from the Oncomine Focus DNA Assay, as described previously [16].

### 2.4. Statistical Analysis

The Fisher and χ2 tests were performed to estimate the associations between MSI status and the somatic mutation profiles or clinical assessments. The Kaplan–Meier method was used to evaluate the cumulative survival probabilities. Statistical differences between survival rates were estimated with the log-rank test. Statistical analysis was performed using MedCalc Statistical Software Version 19.5.3 (MedCalc Software Ltd., Ostend, Belgium). A two-tailed *p* < 0.05 was defined to indicate a statistically significant difference.

## 3. Results

### 3.1. Clinicopathologic Findings of the Patients under Study

The patients under study comprised 50% (24/48) male and 50% (24/48) female Koreans, with a mean age of 64 years (range: 46 to 78). All patients were followed up for at least three years. No disease relapses or GIST-associated deaths occurred during the study period. The clinicopathologic findings of the patients are described in Supplementary Table S1.

### 3.2. Microsatellite Instability Status of Gastrointestinal Stromal Tumors

**Figure 1:**
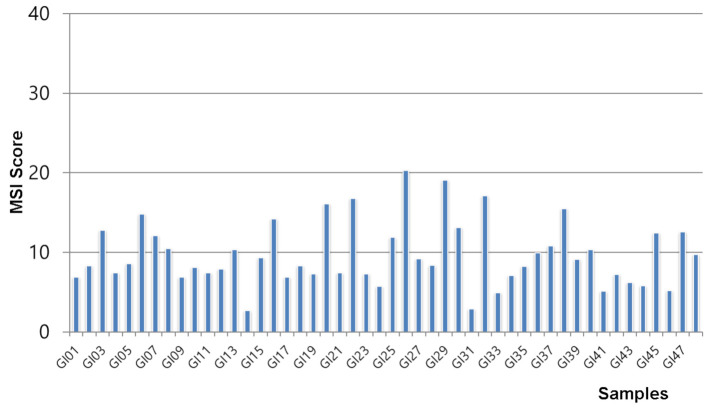
Distribution of microsatellite instability (MSI) scores generated from the Ion AmpliSeq Microsatellite Instability Assay in 48 gastrointestinal stromal tumors.

To examine the MSI status and somatic mutation profiles in the archival FFPE samples, a total of 48 GIST samples were tested in this study. We performed both an Ion AmpliSeq Microsatellite Instability Assay with MSICall and conventional MSI assays to estimate the MSI status in GISTs, and to validate the possibility of NGS-based MSI results and parameter settings. With the Ion AmpliSeq Microsatellite Instability Assay with MSICall, low MSI scores of < 40 were found in all 48 GIST samples, and the average MSI score was 9.7 (range: 2.7 to 20.3) (Figure 1). With the conventional MSI assays, all 48 GIST samples were classified as MSS. As a result, there was 100% (48/48) agreement on MSS between the two MSI assays, but no high or low MSI was observed. Consequently, no associations between MSI status and somatic mutation profiles or clinical assessments were available.

### 3.3. Somatic Mutation Profiles of Gastrointestinal Stromal Tumors

**Figure 2:**
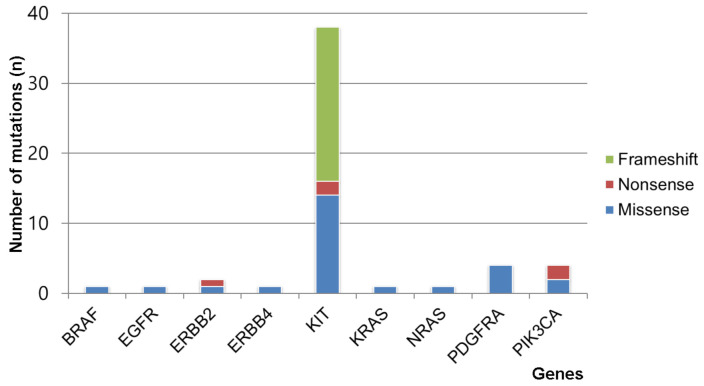
Frequencies of somatic mutation types detected in the various genes by the Oncomine Focus DNA Assay in 48 gastrointestinal stromal tumors. Gene identities are depicted on the x-axis, and the number of mutations is depicted on the y-axis.

A total of 44 (92%) out of the 48 GISTs were from Korean patients; the KIT gene (79%, 38/48) was the most frequently mutated gene, followed by the PDGFRA (8%, 4/48), PIK3CA (8%, 4/48), and ERBB2 (4%, 2/48) mutations. One somatic mutation (2%, 1/48 in each gene) was detected in the BRAF, EGFR, ERBB4, KRAS, and NRAS genes. The mutation distributions of the non-synonymous mutations and small Indels identified in this study are shown in Figure 2.

**Figure 3:**
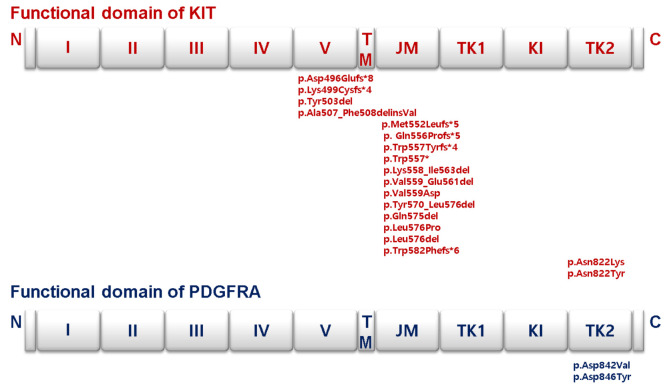
Distribution of somatic mutations in the KIT and PDGFRA functional domains. The somatic mutations in KIT and PDGFRA identified in our study are shown. The types of mutations detected within the domains are indicated above. Boxes represent functional domains: I–V, 5 immunoglobulin-like domain; TM, transmembrane domain; JM, juxtamembrane domain; TK1, tyrosine kinase domain 1; KI, kinase insert domain; TK2, tyrosine kinase domain 2.

The KIT mutation was identified at exon 11 in 31 samples (82%, 31/38), exon 9 in 5 samples (13%, 5/40), and exon 17 in 2 samples (5%, 2/38). On the other hand, the PDGFRA mutation was detected at exon 18 (100%, 4/4) in all four samples (Figure 3). No statistical significance was observed in the disease-free survival analysis among the KIT-mutant (*n* = 38), PDGFRA-mutant (*n* = 4), and wild-type (*n* = 6) GISTs. However, KIT exon 11 mutant patients were more favorable in terms of responding to imatinib than those with exon 9 mutation or wild-type GISTs, and compared to non-KIT exon 11 mutant GISTs (*p* = 0.041).

## 4. Discussion

Solid tumors with dMMR and high MSI carried more missense mutations compared with those with pMMR and MSS [17,18]. Thus, the determination of MSI status has crucially aided treatment plans and clinical prognoses in various solid tumors, and plays a key role in good responses to immunotherapy in solid tumors with dMMR and high MSI [19,20]. However, high MSI status was not shown to be involved in GIST tumorigenesis [1], or in the degree of malignancy and the p53 expression of GISTs [21]. It was also shown that the mechanisms and functions of the MMR system were accomplished in patients with GISTs. Therefore, MSI analysis could not be considered a useful molecular test for estimating immunotherapy response in patients with GISTs. On the contrary, other studies have reported the presence of MSI in 50% (10/22) [22] and 5% (3/62) [23] of patients with GISTs. These discrepancies could be explained by the fact that different molecular tests were used to assess the MSI status, and the analytical sensitivity and specificity of MSI determination depend on the subjective interpretation and methods applied. Consequently, more MSI markers and objective interpretations are required to resolve this controversy about the role of MSI in GISTs. The Ion AmpliSeq Microsatellite Instability Assay used in this study targets 70 mono-base repeats and six di- or tri-base repeats located on 76 microsatellite loci of different chromosomes. The analytical performance of this assay was verified in comparison to the results of conventional MSI analyses for several cancer types [6]. The mapped reads of each marker generated from an NGS-based MSI panel and a bioinformatics pipeline were used to quantify the MSI scores and to objectively determine the MSI status in 48 GISTs. Because MSICall calculated the MSI scores according to targeted microsatellite loci in each sample, this MSI panel sequencing allowed the MSI status of tumor samples to be determined using normal controls as the baseline without matched tumor–normal control samples. It took approximately five days to yield the raw sequencing results. After massively parallel sequencing was completed, the MSICall analysis was performed within one hour. Moreover, MSICall only used the sequencing data of the tumor and baseline control samples, which required less computer resources and could reduce turnaround times. Similarly to previous reports [1,21], all of our GIST patients showed MSS status, which confirmed that MSI status did not participate in the molecular pathogenesis of GISTs. Campanella et al. reported that the features of the MSI phenotype are not sufficient, and this is thus not a biomarker for the immunotherapy response in GISTs [1]. MSI is not associated with the degree of malignancy or the p53 expression of GISTs [21]. Similarly, the absence of cases with a DNA replication phenotype is in keeping with the absence of GISTs in the spectrum of tumors from patients diagnosed with hereditary nonpolyposis colorectal cancer [21]. Interestingly, mitochondrial MSI may play a role, but nuclear MSI may play a smaller role in the development of GISTs, because MSI was detected more frequently in mitochondrial DNA (16%, 10/62) compared to nuclear DNA (5%, 3/62) [23].

On the other hand, most of the GISTs were related to the spontaneous activation of BRAF, KIT, or PDGFRA mutations, along with additional genetic lesions of NF1, as well as components of the succinate dehydrogenase enzymatic complex [24,25]. GISTs commonly carry activated mutations of KIT or PDGFRA genes, and respond strongly to several selective tyrosine kinase inhibitors [26,27]. However, wild-type GISTs harbor no mutations of the KIT or PDGFRA genes, but KIT activation through phosphorylation was still identifiable in these tumors. These could be classified into two groups, according to whether they were deficient or proficient in the succinate dehydrogenase complex. The determination of activated mutations is required to subclassify wild-type GISTs in alternative pathways. To date, optimal systemic therapy for metastatic wild-type GISTs and adjuvant therapy in “high-risk” wild-type GISTs remains questionable, and has no precise guidelines [28]. Consequently, routine genetic mutation profiling could be considered as a crucial issue in the treatment of GISTs when undergoing tyrosine kinase inhibitor therapy [29]. For instance, patients with the exon 11 deletion of the KIT mutations profited from longer durations of adjuvant imatinib. The duration of adjuvant imatinib treatment changed the risk of GIST relapse related to some KIT mutations, such as deletions that affected exon 11 codons 557 and/or 558 [28]. Moreover, the achievement of secondary mutations in either KIT or PDGFRA revealed the most common mechanism of imatinib resistance in GISTs [30]. Similarly to our previous study [16], this study also confirmed the usefulness of targeted NGS with a cancer gene panel for efficiently detecting somatic mutations related to GISTs.

## 5. Conclusions

In conclusion, an NGS-based MSI panel with MSICall confirmed a rare phenomenon of microsatellite instability in GISTs, irrespective of diverse genomic alterations. The Ion AmpliSeq Microsatellite Instability Assay could prove to be a reliable streamline test for MSI determination beyond conventional MSI assays in GISTs, as well as several types of solid tumors. Massively parallel sequencing can simultaneously determine MSI status and the somatic mutation profile in a single test. This combined approach may help us to understand the molecular pathogenesis of GIST carcinogenesis and malignant progression.

## Data Availability

The data presented in this study are available on request from the corresponding author.

## Supplementary Materials

The following are available online at https://www.mdpi.com/1010-660X/57/2/174/s1, Table S1: Clinicopathologic findings in 48 Korean patients with gastrointestinal stromal tumors.

## References

[1] Campanella N.C., Scapulatempo-Neto C., Abrahão-Machado L.F., De Oliveira A.T.T., Berardinelli G.N., Guimarães D.P., Reis R.M. (2017). Lack of microsatellite instability in gastrointestinal stromal tumors. Oncol. Lett..

[2] Chapusot C., Martin L., Bouvier A.M., Bonithon-Kopp C., Ecarnot-Laubriet A., Rageot D., Ponnelle T., Laurent-Puig P., Faivre J., Piard F. (2002). Microsatellite instability and intratumoural heterogeneity in 100 right-sided sporadic colon carcinomas. Br. J. Cancer.

[3] Palmieri G., Colombino M., Cossu A., Marchetti A., Botti G., Ascierto P.A. (2017). Genetic instability and increased mutational load: Which diagnostic tool best direct patients with cancer to immunotherapy?. J. Transl. Med..

[4] Watson N., Grieu F., Morris M., Harvey J., Stewart C., Schofield L., Goldblatt J., Iacopetta B. (2007). Heterogeneous Staining for Mismatch Repair Proteins during Population-Based Prescreening for Hereditary Nonpolyposis Colorectal Cancer. J. Mol. Diagn..

[5] Shah S.N., Hile S.E., Eckert K.A. (2010). Defective Mismatch Repair, Microsatellite Mutation Bias, and Variability in Clinical Cancer Phenotypes. Cancer Res..

[6] Hirotsu Y., Nagakubo Y., Amemiya K., Oyama T., Mochizuki H., Omata M. (2020). Microsatellite Instability Status is De-termined by Targeted Sequencing with MSIcall in 25 Cancer Types. Clin. Chim. Acta.

[7] Hause R.J., Pritchard C.C., Shendure R.J.H.J., Salipante C.C.P.S.J. (2016). Classification and characterization of microsatellite instability across 18 cancer types. Nat. Med..

[8] Bonneville R., Krook M.A., Kautto E.A., Miya J., Wing M.R., Chen H.-Z., Reeser J.W., Yu L., Roychowdhury S. (2017). Landscape of Microsatellite Instability Across 39 Cancer Types. JCO Precis. Oncol..

[9] (2014). The Cancer Genome Atlas Research Network Comprehensive molecular characterization of gastric adenocarcinoma. Nat. Cell Biol..

[10] Kim S.Y., Choi Y.Y., An J.Y., Shin H.B., Jo A., Choi H., Seo S.H., Bang H.-J., Cheong J.-H., Hyung W.J. (2015). The benefit of microsatellite instability is attenuated by chemotherapy in stage II and stage III gastric cancer: Results from a large cohort with subgroup analyses. Int. J. Cancer.

[11] Polom K., Marrelli D., Pascale V., Ferrara F., Voglino C., Marini M., Roviello F. (2017). The pattern of lymph node metastases in microsatellite unstable gastric cancer. Eur. J. Surg. Oncol. (EJSO).

[12] Smyth E.C., Wotherspoon A., Peckitt C., Gonzalez D., Hulkki-Wilson S., Eltahir Z., Fassan M., Rugge M., Valeri N., Okines A. (2017). Mismatch Repair Deficiency, Microsatellite Instability, and Survival. JAMA Oncol..

[13] Choi Y.Y., Kim H., Shin S.-J., Kim H.Y., Lee J., Yang H.-K., Kim W.H., Kim Y.-W., Kook M.-C., Park Y.K. (2019). Microsatellite Instability and Programmed Cell Death-Ligand 1 Expression in Stage II/III Gastric Cancer. Ann. Surg..

[14] Kang Y.-K., Kang H.J., Kim K.-M., Sohn T., Choi N., Ryu M.-H., Kim W.H., Yang H.-K. (2012). Clinical Practice Guideline for Accurate Diagnosis and Effective Treatment of Gastrointestinal Stromal Tumor in Korea. Cancer Res. Treat..

[15] Soreide K. (2011). High-fidelity of five quasimonomorphic mononucleotide repeats to high-frequency microsatellite in-stability distribution in early-stage adenocarcinoma of the colon. Anticancer Res..

[16] Park J., Lee S.-I., Shin S., Hong J.H., Yoo H.M., Kim J.G. (2020). Genetic profiling of somatic alterations by Oncomine Focus Assay in Korean patients with advanced gastric cancer. Oncol. Lett..

[17] Chalmers Z.R., Connelly C.F., Fabrizio D., Gay L., Ali S.M., Ennis R., Schrock A., Campbell B., Shlien A., Chmielecki J. (2017). Analysis of 100,000 human cancer genomes reveals the landscape of tumor mutational burden. Genome Med..

[18] Fabrizio D.A., Jr T.J.G., Dunne R.F., Frampton G., Sun J., Gowen K., Kennedy M., Greenbowe J., Schrock A.B., Hezel A.F. (2018). Beyond microsatellite testing: Assessment of tumor mutational burden identifies subsets of colorectal cancer who may respond to immune checkpoint inhibition. J. Gastrointest. Oncol..

[19] Le D.T., Uram J.N., Wang H., Bartlett B.R., Kemberling H., Eyring A.D., Skora A.D., Luber B.S., Azad N.S., Laheru D. (2015). PD-1 Blockade in Tumors with Mismatch-Repair Deficiency. N. Engl. J. Med..

[20] Le D.T., Durham J.N., Smith K.N., Wang H., Bartlett B.R., Aulakh L.K., Lu S., Kemberling H., Wilt C., Luber B.S. (2017). Mismatch repair deficiency predicts response of solid tumors to PD-1 blockade. Science.

[21] Lopes J.M., Silva P., Seixas M., Cirnes L., Seruca R. (1998). Microsatellite instability is not associated with degree of malignancy and p53 expression of gastrointestinal stromal tumours. Histopathology.

[22] Fukasawa T., Chong J.-M., Sakurai S., Koshiishi N., Ikeno R., Tanaka A., Matsumoto Y., Hayashi Y., Koike M., Fukayama M. (2000). Allelic Loss of 14q and 22q, NF2Mutation, and Genetic Instability Occur Independently of c-kitMutation in Gastrointestinal Stromal Tumor. Jpn. J. Cancer Res..

[23] Kose K., Hiyama T., Tanaka S., Yoshihara M., Yasui W., Chayama K. (2006). Nuclear and Mitochondrial DNA Microsatellite Instability in Gastrointestinal Stromal Tumors. Pathobiology.

[24] El-Menyar A., Mekkodathil A., Al-Thani H. (2017). Diagnosis and management of gastrointestinal stromal tumors: An up-to-date literature review. J. Cancer Res. Ther..

[25] Charville G.W., Longacre T.A. (2017). Surgical Pathology of Gastrointestinal Stromal Tumors: Practical Implications of Morphologic and Molecular Heterogeneity for Precision Medicine. Adv. Anat. Pathol..

[26] Szucs Z., Thway K., Fisher C., Bulusu R., Constantinidou A., Benson C., Van Der Graaf W.T.A., Jones R.L. (2017). Molecular subtypes of gastrointestinal stromal tumors and their prognostic and therapeutic implications. Future Oncol..

[27] Mei L., Du W., Idowu M., Von Mehren M., Boikos S.A. (2018). Advances and Challenges on Management of Gastrointestinal Stromal Tumors. Front. Oncol..

[28] Joensuu H., Wardelmann E., Sihto H., Eriksson M., Hall K.S., Reichardt A., Hartmann J.T., Pink D., Cameron S., Hohenberger P. (2017). Effect of KIT and PDGFRA Mutations on Survival in Patients With Gastrointestinal Stromal Tumors Treated With Adjuvant Imatinib: An Exploratory Analysis of a Randomized Clinical Trial. JAMA Oncol..

[29] Yamamoto H., Oda Y. (2014). Gastrointestinal stromal tumor: Recent advances in pathology and genetics. Pathol. Int..

[30] Heinrich M.C., Maki R.G., Corless C.L., Antonescu C.R., Harlow A., Griffith D., Town A., McKinley A., Ou W.-B., Fletcher J.A. (2008). Primary and Secondary Kinase Genotypes Correlate With the Biological and Clinical Activity of Sunitinib in Imatinib-Resistant Gastrointestinal Stromal Tumor. J. Clin. Oncol..

