# Supplementary material for: Rare Occurrence of Microsatellite Instability in Gastrointestinal Stromal Tumors
Source: Medicina (Kaunas). 2021 Feb 18;57(2):174. doi: 10.3390/medicina57020174 (PMC7931009; doi:10.3390/medicina57020174)
Supplement: Supplementary file 1 [file medicina-57-00174-s001.pdf]

Supplementary Table S1. Clinicopathologic findings in 48 Korean patients with gastrointestinal stromal tumors

Patient	Sex /Age(yr)	Size (cm)	Mitosis (/50HPF)	Risk assessment	Cellularity	Pleomorphism	Necrosis	Invasion	Immunohistochemistry*		
									Actin	CD34	Ki-67
DJGST01	M/60	2.9	4	Low	Mild	Mild	Neg	Neg	Pos	Pos	Pos
DJGST02	M/63	4.7	7	Intermediate	Moderate	Moderate	Pos	Neg	Neg	Pos	Pos
DJGST03	F/51	1.7	1	Very low	Mild	Mild	Neg	Neg	Neg	Pos	Neg
DJGST04	M/66	3.9	6	Intermediate	Mild	Moderate	Pos	Neg	Neg	Pos	Pos
DJGST05	F/59	2.3	3	Low	Mild	Mild	Neg	Neg	Pos	Neg	Pos
DJGST06	M/62	1.9	1	Very low	Mild	Mild	Neg	Neg	Neg	Pos	Pos
DJGST07	F/46	2.8	4	Low	Mild	Mild	Neg	Neg	Neg	Pos	Neg
DJGST08	M/72	3.2	4	Low	Mild	Mild	Pos	Neg	Neg	Pos	Neg
DJGST09	F/71	1.8	2	Very low	Mild	Mild	Neg	Neg	Neg	Pos	Neg
DJGST10	F/62	3.4	3	Low	Mild	Mild	Neg	Neg	Neg	Pos	Pos
DJGST11	F/78	4.1	7	Intermediate	Mild	Moderate	Neg	Neg	Neg	Pos	Neg
DJGST12	M/61	4.7	5	Intermediate	Moderate	Mild	Neg	Neg	Neg	Pos	Pos
DJGST13	M/61	2.8	3	Low	Mild	Mild	Neg	Neg	Pos	Pos	Pos
DJGST14	F/57	3.9	7	Intermediate	Moderate	Moderate	Neg	Mucosal	Pos	Pos	Pos
DJGST15	F/57	1.7	1	Very low	Mild	Mild	Neg	Neg	Pos	Pos	Neg
DJGST16	M/63	2.9	3	Low	Moderate	Mild	Neg	Mucosal	Neg	Pos	Pos
DJGST17	M/56	2.8	4	Low	Moderate	Moderate	Neg	Neg	Neg	Pos	Pos
DJGST18	M/63	5.2	6	Intermediate	Moderate	Moderate	Pos	Neg	Pos	Pos	Pos
DJGST19	M/67	3.3	2	Low	Mild	Mild	Neg	Neg	Neg	Pos	Neg
DJGST20	F/63	1.5	2	Very low	Mild	Mild	Neg	Neg	Neg	Pos	Neg
DJGST21	F/53	3.9	5	Intermediate	Moderate	Moderate	Neg	Perineural	Neg	Pos	Pos
DJGST22	F/49	2.3	3	Low	Mild	Mild	Neg	Neg	Neg	Pos	Neg
DJGST23	F/74	6.4	>10	High	Moderate	Moderate	Neg	Neg	Neg	Pos	Pos
DJGST24	M/67	4.8	6	Intermediate	Moderate	Mild	Neg	Perineural	Neg	Pos	Pos
DJGST25	F/73		9	High	Moderate	Moderate	Pos	Mucosal	Neg	Neg	Pos
DJGST26	F/74	3.4	6	Intermediate	Severe	Mild	Neg	Mucosal	Neg	Pos	Pos
DJGST27	M/57	5.1	7	Intermediate	Mild	Moderate	Neg	Neg	Pos	Pos	Pos
DJGST28	M/71	1.9	1	Very low	Mild	Mild	Neg	Neg	Neg	Pos	Neg
DJGST29	F/62	7.4	>10	High	Severe	Moderate	Pos	Neg	Neg	Neg	Pos
DJGST30	M/74	2.8	3	Low	Mild	Mild	Neg	Neg	Neg	Neg	Neg

DJGST31	F/61	3.4	7	Intermediate	Moderate	Moderate	Pos	Neg	Pos	Pos	Neg
DJGST32	M/72	6.5	>10	High	Severe	Moderate	Pos	Neg	Pos	Pos	Pos
DJGST33	M/74	2.8	3	Low	Mild	Mild	Neg	Neg	Neg	Neg	Neg
DJGST34	F/60	4.6	6	Intermediate	Moderate	Moderate	Neg	Neg	Pos	Pos	Neg
DJGST35	M/59	6.6	9	High	Moderate	Mild	Neg	Perineural, LN&Vas	Pos	Pos	Pos
DJGST36	F/72	3.1	3	Low	Mild	Mild	Neg	Neg	Pos	Pos	Neg
DJGST37	F/59	5.5	6	Intermediate	Moderate	Mild	Neg	Neg	Pos	Pos	Pos
DJGST38	F/72	2.6	3	Low	Mild	Mild	Neg	Neg	Neg	Pos	Pos
DJGST39	F/62	3.2	3	Low	Moderate	Moderate	Neg	Neg	Pos	Pos	Neg
DJGST40	M/67	5.5	9	High	Severe	Moderate	Neg	Neg	Neg	Pos	Pos
DJGST41	F/73	9.7	>10	High	Severe	Moderate	Neg	Mucosal	Neg	Pos	Pos
DJGST42	M/62	2.8	3	Low	Mild	Mild	Neg	Neg	Neg	Pos	Neg
DJGST43	M/74	2.5	2	Low	Moderate	Moderate	Neg	Mucosal	Neg	Neg	Pos
DJGST44	M/60	4.7	6	Intermediate	Moderate	Moderate	Neg	Neg	Pos	Pos	Neg
DJGST45	M/61	3.9	5	Intermediate	Moderate	Mild	Pos	Neg	Pos	Pos	Neg
DJGST46	M/58	2.7	3	Low	Moderate	Mild	Neg	Neg	Neg	Pos	Pos
DJGST47	F/76	6.7	>10	High	Moderate	Moderate	Pos	Neg	Neg	Pos	Pos
DJGST48	F/59	2.9	4	Low	Moderate	Mild	Neg	Neg	Neg	Pos	Neg

LN&Vas, lymphnode & vascular

*All 48 patients positive for c-KIT but not for S100 and desmin were diagnosed as GIST.
